# Detection of Aberrant Glycosylation of Serum Haptoglobin for Gastric Cancer Diagnosis Using a Middle-Up-Down Glycoproteome Platform

**DOI:** 10.3390/jpm11060575

**Published:** 2021-06-18

**Authors:** Seunghyup Jeong, Unyong Kim, Myungjin Oh, Jihyeon Nam, Sehoon Park, Yoonjin Choi, Dongho Lee, Jaehan Kim, Hyunjoo An

**Affiliations:** 1 Asia-Pacific Glycomics Reference Site, Chungnam National University, Daejeon 34134, Korea; shjeong0512@cnu.ac.kr (S.J.); mjoh@cnu.ac.kr (M.O.); namjihyeon97@o.cnu.ac.kr (J.N.); 2 Graduate School of Analytical Science and Technology, Chungnam National University, Daejeon 34134, Korea; 3 Biocomplete Inc., Seoul 08389, Korea; unyong.kim@biocomplete.co.kr; 4 Division of Hematology/Oncology, Department of Medicine, Samsung Medical Center, Sungkyunkwan University School of Medicine, Seoul 06351, Korea; hematoma@skku.edu; 5 Department of Internal Medicine, Yonsei University College of Medicine, Seoul 03722, Korea; erica0007@gmail.com; 6 Department of Internal Medicine for Gastroenterology, Seoul National University Bundang Hospital, Seongnam 13620, Korea; dhljohn@snubh.org; 7 Department of Food and Nutrition, Chungnam National University, Daejeon 34134, Korea; jaykim@cnu.ac.kr

**Keywords:** gastric cancer, middle-up-down, haptoglobin, glycopeptide, biomarker, mass spectrometry

## Abstract

Gastric cancer is a frequently occurring cancer and is the leading cause of cancer-related deaths. Recent studies have shown that aberrant glycosylation of serum haptoglobin is closely related to gastric cancer and has enormous potential for use in diagnosis. However, there is no platform with high reliability and high reproducibility to comprehensively analyze haptoglobin glycosylation covering microheterogeneity to macroheterogeneity for clinical applications. In this study, we developed a middle-up-down glycoproteome platform for fast and accurate monitoring of haptoglobin glycosylation. This platform utilizes an online purification of LC for sample desalting, and an in silico haptoglobin glycopeptide library constructed by combining peptides and N-glycans to readily identify glycopeptides. In addition, site-specific glycosylation with glycan heterogeneity can be obtained through only a single MS analysis. Haptoglobin glycosylation in clinical samples consisting of healthy controls (*n* = 47) and gastric cancer patients (*n* = 43) was extensively investigated using three groups of tryptic glycopeptides: GP1 (including Asn184), GP2 (including Asn207 and Asn211), and GP3 (including Asn241). A total of 23 individual glycopeptides were determined as potential biomarkers (*p* < 0.00001). In addition, to improve diagnostic efficacy, we derived representative group biomarkers with high AUC values (0.929 to 0.977) through logistic regression analysis for each GP group. It has been found that glycosylation of haptoglobin is highly associated with gastric cancer, especially the glycosite Asn241. Our assay not only allows to quickly and easily obtain information on glycosylation heterogeneity of a target glycoprotein but also makes it an efficient tool for biomarker discovery and clinical diagnosis.

## 1. Introduction

Gastric cancer caused by genetic factors, dietary habits, smoking, alcohol consumption, and infection with *Helicobacter pylori* is one of the most frequently occurring cancer, with approximately 1,000,000 new cases each year and more than 750,000 deaths worldwide [1,2,3]. Serum protein markers, such as CEA (carcinoembryonic antigen), CA19-9 (carbohydrate antigen 19-9), CA72-4 (carbohydrate antigen 72-4), and CA125 (carbohydrate antigen 125) have been widely used in clinical practice for gastric cancer detection [4,5]. However, since these serological markers do not have sufficient sensitivity and specificity, an apparent but invasive gastroscopy is often used for gastric cancer diagnosis. Therefore, there is a need in clinic for a new analytical platform that is fast, accurate, and non-invasive with high sensitivity and high specificity [6].

Glycosylation is one of the most important post-translational modifications (PTMs) and plays a pivotal role in various biological processes, such as protein function and cell–cell interaction [7,8]. In addition, glycosylation has great potential as a biomarker for cancer and infectious diseases because it is highly sensitive to biological environments [9]. Therefore, studies based on serum and cell glycomic profiling have been conducted for biomarker discovery and cancer diagnosis [10,11,12]. In particular, cancer progression, including angiogenesis, cell–cell adhesion, and tumor metastasis, is known to be associated with glycosylation in various cancers [13,14,15]. For example, high mannose type N-glycans have been found to be specific molecules in breast cancer patients, and sialylated O-glycans, such as Tn antigen, sialyl- Le^x^, and sialyl-Le^a^, on the surface of tumor cells are known to be one of the important molecules for metastasis [16,17,18,19,20]. Recently, glyco-biomarker studies are moving towards in-depth glycosylation characterization of a target glycoprotein that can improve diagnostic specificity and sensitivity for efficient clinical applications [21,22,23,24].

Haptoglobin is one of the major serum components that accounts for 0.4–2.6% of total blood proteins, and a highly sialylated glycoprotein containing four N-glycosylation sites (Asn184, 207, 211, and 241) on *β*-subunit [25], whose glycosylation changes in several types of cancer, such as hepatic, prostate, ovarian, and pancreatic [26,27,28,29]. Interestingly, recent studies clearly suggest that there is an overt correlation between aberrant glycosylation of haptoglobin and gastric cancer through glycomic and glycoproteomic approaches [30,31,32,33]. Despite the tremendous potential of haptoglobin glycosylation as a cancer biomarker, there is no clinically compatible assay platform that offers high reliability and reproducibility based on extensive characterization of haptoglobin glycosylation, including the distribution of glycans present at a specific site (microheterogeneity) and the occupancy of glycans at individual sites (macroheterogeneity) [34].

Mass spectrometry (MS) is a powerful tool used in various omics, from proteomics to glycomics [35,36,37]. The introduction of MS in glycomics has accelerated the study of biomarkers [38,39,40,41,42]. Initially, biomarkers of several types of diseases, particularly immune-related diseases and cancers, have been successfully determined through overall glycan profiling [43,44,45,46,47,48]. In recent years, the target of analysis is shifting from the conventional global glycan profiling to the target glycoprotein, and various MS-based analytical tools have been developed to monitor abnormal glycosylation of a glycoprotein. It ranges from glycopeptide analysis, which provides information about site-specific glycosylation, to complete intact glycoprotein analysis, which provides intuitive information on the degree of glycosylation of the whole protein [31,32,49,50,51]. In particular, glycoproteomic analysis at the glycopeptide level is an effective method that can simultaneously acquire information on glycans and glycosylation sites [52,53,54], but it is not utilized in clinics due to the time required for the sample preparation step, difficulties of tandem MS analysis, and complex data interpretation. Therefore, there is a need for an easy and fast glycoproteomic analysis tool that can be used in the clinical field [7].

In this study, we developed a new middle-up-down glycoproteome platform that can quickly and accurately monitor the abnormal glycosylation on the target glycoprotein, haptoglobin, for the diagnosis of gastric cancer. With this approach, samples can be purified online in LC and then directly separated and detected on a diphenyl column without further purification of the glycopeptide, resulting in faster sample analysis. In addition, the in silico glycopeptide library allowed us to obtain information ranging from microheterogeneity to macroheterogeneity of the target glycoprotein without tandem MS analysis. For biomarker discovery, a middle-up-down glycoproteome platform was applied to gastric cancer patients (*n* = 43) and healthy controls (*n* = 47), and apparent differences in glycosylation were found even in a small sample set. A total of 23 individual glycopeptide biomarkers that were statistically significant were determined, which showed high sensitivity and specificity (AUC 0.783 to 0.901). Interestingly, most of the markers were complex type N-glycan decorated with sialylation, of which more than half were simultaneously fucosylated. In addition, potential biomarkers were classified into three groups according to the peptide sequence to determine the biological association between haptoglobin glycosylation heterogeneity and gastric cancer, indicating that the glycosite Asn241 is more closely related. The middle-up-down glycoproteoform approach enables easy and fast analysis of site-specific glycosylation as well as glycan heterogeneity for specific target glycoprotein, making it a powerful platform for biomarker discovery and diagnosis through large clinical samples.

## 2. Materials and Methods

### 2.1. Materials and Reagents

Commercial human serum, ammonium bicarbonate (NH_4_HCO_3_), and iodoacetamide (IAA) were purchased from Sigma-Aldrich (St. Louis, MO, USA). Sequencing grade modified trypsin and dithiothreitol (DTT) were purchased from Promega (Madison, WI, USA). Anti-human haptoglobin was obtained from Dako (Carpinteria, CA, USA). All other solvents used in LC–MS analysis were purchased from Sigma-Aldrich, which were analytical grade or higher.

### 2.2. Serum Samples from Gastric Cancer Patients and Healthy Control Subjects

The clinical information of the serum samples is summarized in Table S1. A total of 90 serum samples were used, and the population consisted of 47 healthy controls and 43 gastric cancer patients (Stage IV, adenocarcinoma type). The research design and protocol were reviewed and approved by the Institutional Review Board of the participating hospital, the Samsung Medical Center, Seoul, Republic of Korea (IRB# SMC2015-07-146-001). Cancer diagnoses and stage determinations were examined based on endoscopic ultrasound, biopsy, and gastrectomy for each patient. All participating subjects, including the healthy control, were Korean and provided informed consent for obtaining the serum samples. The samples were stored at −80 °C until further processing.

### 2.3. Haptoglobin Purification from Serum Samples

Serum haptoglobin was purified using an anti-haptoglobin immunoaffinity column as described in a previous study [30]. In brief, 450 μL serum from each sample subject was diluted with 4.5 mL phosphate-buffered saline (PBS: 10 mM phosphate buffer, 2.7 mM potassium chloride, 137 mM sodium chloride, pH 7.4), and then applied to the anti-haptoglobin immunoaffinity column. After a binding reaction, the unbound components were washed by 30 mL PBS, the haptoglobin was eluted with elution buffer (0.1 M glycine, 0.5 M NaCl, pH 2.8), and the eluent was fractionated into a tube containing neutralization buffer (1.0 M Tris-HCl, pH 9.0). A centrifugal filter (MWCO 10,000, Amicon Ultra, Millipore; Billerica, MA, USA) was used to remove the detergent from the eluent and the quantification of the purified haptoglobin was assayed using a Quanti-iT Assay Kit (Invitrogen; Carlsbad, CA, USA). To confirm the purity of the haptoglobin, the eluent was randomly applied to 12.5% SDS-PAGE with Coomassie Brilliant Blue staining. Each purified sample was lyophilized and kept at −80 °C until enzymatic digestion.

### 2.4. Enzymatic Digestion for Glycopeptide Production

The purified serum haptoglobin (20 μg) was dissolved in a buffer, which consisted of 50 mM NH_4_HCO_3_ and 10 mM DTT. The haptoglobin dissolved in the buffer solution was placed in a 95 °C water for 10 min to reduce the disulfide bond and separate the *α*- and *β*-subunits of the haptoglobin, and then alkylated with 50 mM IAA to prevent reassembly of the disulfide bond. Finally, trypsin was added to the digestion and the mixture was incubated in a 37 °C water bath for 16 h.

### 2.5. LC–MS Analysis of Haptoglobin Glycopeptide

After trypsin digestion, 6.0 μL of haptoglobin peptides (corresponding to 2 μg protein) were directly injected by an autosampler into the LC–MS system, which consisted of a 6550 iFunnel Q-TOF coupled to a 1290 Infinity II UHPLC system (Agilent Technologies, San Jose, CA, USA). First, the samples were desalted on a 2.1 × 12.5 mm narrow-bore C8 guard column and delivered to a 2.1 × 100 mm Rapid Resolution High Definition (RRHD) diphenyl column (Agilent Technologies) for separation. A rapid elution gradient for haptoglobin peptides was applied at 200 μL/min using mobile phases of (A) 0.3% formic acid in nanopure water, and (B) 0.3% formic acid in acetonitrile, ramping up from 5 to 95% over the course of 27 min. The column was flushed with 95% solvent B for 10 min and then re-equilibrated for 3 min before analyzing the next sample. The column temperature was maintained at 30 °C during the analysis. Following LC separation, the haptoglobin peptides were ionized and detected on the positive ion mode over a mass range of m/z 500 to 3200, with an acquisition rate of 2 spectra per second.

### 2.6. Data Processing, Glycopeptide Identification, and Statistical Analysis

All raw LC–MS data were processed by a molecular feature extraction algorithm included in the MassHunter Qualitative Analysis software (version B.07.00 SP1, Agilent Technologies). MS peaks were filtered with a signal-to-noise ratio of 5.0 and glycopeptide compounds were founded from deconvoluted masses by the theoretical accurate mass of the in silico haptoglobin glycopeptide library with a 10 ppm mass tolerance. The in silico glycopeptide library was built by the combination of theoretical tryptic peptides of haptoglobin and N-glycans obtained experimentally. An individual *t*-test analysis by Microsoft Excel 2016 (Microsoft, Seattle, WA, USA) was used to identify the statistical differences between gastric cancer patients and healthy controls, and all *p*-values were applied with a two-tailed analysis. The receiver operation characteristic (ROC) curve of each potential glycopeptide biomarker and logistic regression analysis for the combined biomarkers were performed by IBM SPSS Statistics (version 24, IBM, Armonk, NY, USA). Hierarchical Clustering Explorer (version 3.5, HCIL, University of Maryland, College Park, MD, USA) was used to confirm the reproducibility.

## 3. Results and Discussion

### 3.1. Analytical Strategy Using a Middle-Up-Down Glycoproteome Approach

**Figure 1:**
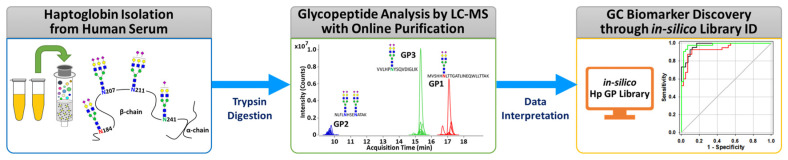
The overall experimental workflow of the middle-up-down glycoproteomic approach for aberrant glycosylation monitoring and gastric cancer diagnosis.

The overall experimental workflow for middle-up-down glycoproteomic analysis is shown in Figure 1: (i) purification of the targeted serum haptoglobin via immunoaffinity chromatography; (ii) trypsin treatment of haptoglobin for glycopeptide generation; (iii) glycopeptide profiling via UHPLC Q-TOF MS with online purification; (iv) glycopeptide identification through in silico haptoglobin library; (v) biomarker discovery by a group of glycopeptides based on glyco-heterogeneity. The most notable points of this platform are three things. First, haptoglobin glycopeptides were injected into LC–MS without further enrichment and purification and desalted directly online through a C8-packed guard column by a quaternary pump, and then transferred to the diphenyl analytical column by valve switching [31]. This online enrichment allows the analysis of large numbers of samples with minimal preparation steps and saves time, cost, and labor, which are important considerations in clinical applications. Second, the glycan microheterogeneity and macroheterogeneity of the haptoglobin were quickly and accurately monitored through tryptic glycopeptides analyzed by only a single MS. We created an in silico haptoglobin glycopeptide library to facilitate the identification of glycopeptide by combining the theoretical tryptic peptides of haptoglobin with the experimentally obtained mass values of 41 N-glycans (Table S2). Site-specific glycopeptides, including four individual glycosylation sites via trypsin treatment, cannot be fully produced compared with a multiple enzyme reaction or non-specific protease treatment. However, this approach has the distinct advantage of significantly reducing the time, cost, and labor required for sample preparation, and further simplifying data processing by facilitating the identification of glycopeptides of Hp via an in silico library. Finally, unlike the discovery of individual molecular markers in typical biomarker studies, we reclassified individual potential markers according to their glycosylation site and determined group biomarkers to monitor changes in the glycosylation site of haptoglobin in gastric cancer. Group-by-group analysis of glycopeptides not only enables efficient detection of gastric cancer markers but also provides information on the glycosylation site that is biologically more sensitive to gastric cancer. In summary, the middle-up-down glycoproteomic platform analyzes the target glycoprotein at the glycopeptide level (middle-up) with minimal sample preparation and single MS analysis and also provides glyco-heterogeneity information (middle-down) as well as biological links between cancer and glycosylation sites.

Prior to the analysis of clinical samples for biomarker discovery, the reproducibility of our platform was validated with standard haptoglobin purified from a commercial human serum. As shown in Figure S1, the Pearson correlation coefficient (*R*) values for the comparison of replicates were 1.000 for GP1 (16 pairs) and GP3 (19 pairs), and from 0.984 to 0.998 for GP2 (36 pairs), indicating high reproducibility and reliability.

### 3.2. Identification of Glycopeptides Using In Silico Haptoglobin Glycopeptide Library

**Figure 2:**
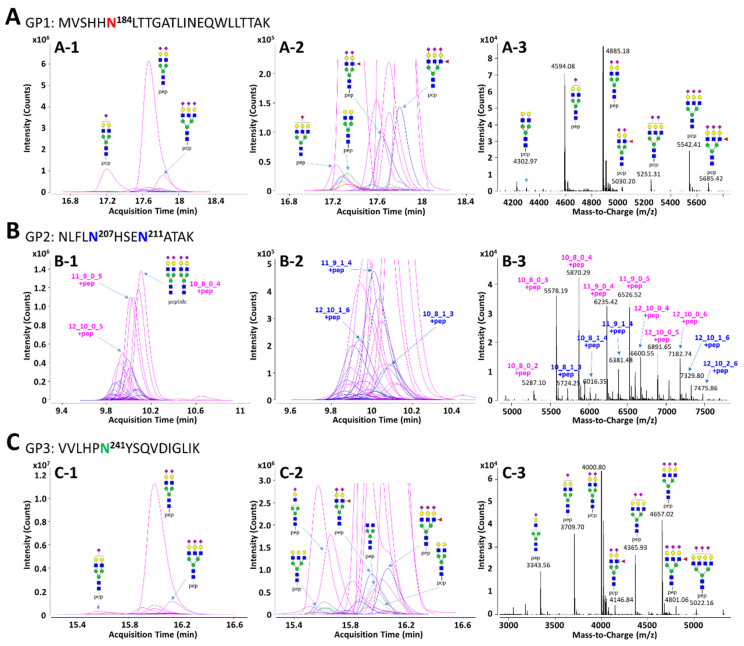
Representative extracted compound chromatograms (ECCs) and deconvoluted spectra of GP1 (**A**), GP2 (**B**), and GP3 (**C**) of haptoglobin purified from a commercial serum. The peptide sequence of each GP is inserted in the figure. The left column, (**A-1**), (**B-1**), and (**C-1**), represents the ECCs of Hp glycopeptides identified using the in silico haptoglobin glycopeptide library. The middle column, (**A-2**), (**B-2**), and (**C-2**), shows magnified views of low abundant glycopeptides on ECCs. The right column, (**A-3**), (**B-3**), and (**C-3**), represents deconvoluted spectra of the retention time range that glycopeptides are eluted. Glycans on GP2 were represented by composition rather than structure since there are two glycosylation sites in one peptide. For feasible interpretation and visualization of low abundant glycans, the *y*-axes of GP1 and GP3 are displayed at about 3× magnification. Purple color—only sialylation; blue color—fucosylation and sialylation.

**Figure 3:**
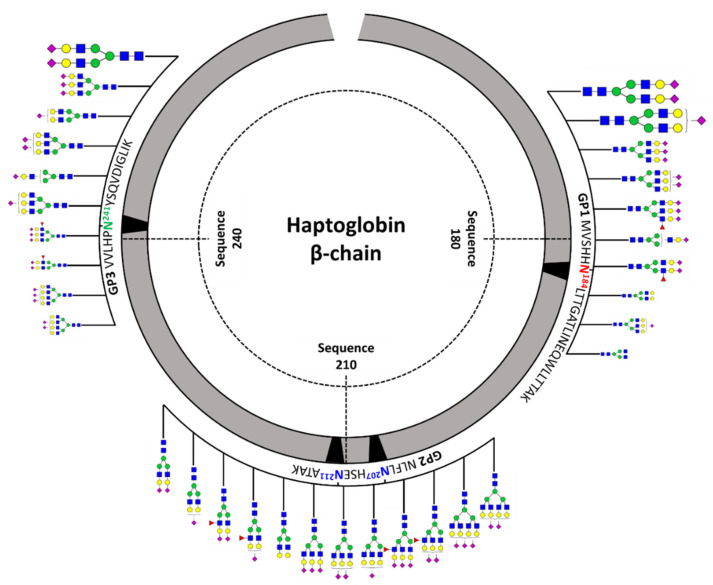
Site-specific glycosylation mapping of the haptoglobin standard purified from commercial human sera. For GP1 and GP3, the top 10 representative major glycoforms for each glycosylation site were indicated. Bi-antennary di-sialylated N-glycan occupies more than 50% of the total for each site. The sizes of N-glycan cartoon on GP1 and GP3 represent their relative abundance in each glycopeptide group. The biggest is more than 10%; medium is more than 1%; the smallest is less than 1%. In GP2 with two glycosylation sites, the denoted N-glycan cartoon is the composition most probable to constitute the top 10 glycopeptides of GP2 based on the abundance of haptoglobin glycan profiling.

In order to facilitate the interpretation of MS data and to efficiently identify glycopeptides, the in silico haptoglobin glycopeptide library was constructed by the combination of haptoglobin N-glycans obtained experimentally and the theoretical mass of tryptic peptides. The top 41 N-glycans, accounting for 99% of the total haptoglobin N-glycan quantity, were used for the library [30]. The peptide sequence of haptoglobin was referenced from the UniProt human protein database (P00738) and the theoretical mass of haptoglobin peptides was calculated using PeptideMass tool of ExPASy (https://www.expasy.org/, accessed on 9 December 2020). The amino acid sequence of the protein may be generated isoform by alternative splicing [55], but the isoform of haptoglobin does not change the sequence of *β*-subunit where the glycosylation site exists. Therefore, the in silico haptoglobin library was constructed using only a canonical sequence. Four potential N-glycosylation sites in the *β*-subunit of haptoglobin were divided into groups of three kinds of tryptic glycopeptide classified by the same peptide sequence: GP1 (Asn184, MVSHHN^184^LTTGATLINEQWLLTTAK), GP2 (Asn207 and Asn211, NLFLN^207^HSEN^211^ATAK), and GP3 (Asn241, VVLHPN^241^YSQVDIGLIK). Based on 41 N-glycan compositions, each GP1 and GP3 with one glycosylation site could have 41 possible glycoforms. Since GP2 has two glycosylation sites, Asn207 and Asn211, there are theoretically 902 glycoforms possible. However, if the total composition of N-glycans that may be present in the two glycosylation sites is the same (e.g., Asn207-Hex_6_HexNAc_5_Fuc_1_NeuAc_3_ + Asn211-Hex_5_HexNAc_4_NeuAc_2_ and Asn207-Hex_5_HexNAc_4_Fuc_1_NeuAc_2_ + Asn211-Hex_6_HexNAc_5_NeuAc_3_), they were recognized as duplicates and excluded from the glycopeptide library. As a result, GP2 could have a total of 416 possible glycoforms in the library. The full list of the in silico haptoglobin glycopeptide library is provided in Table S3. Representative extracted compound chromatograms (ECCs) and their mass spectra of three glycopeptide groups of haptoglobin purified from a commercial serum are shown in Figure 2. Glycopeptide groups were detected in the order of GP2, GP3, and GP1, and the elution times of each group were completely separated without overlapping. Each elution time was 10 to 11 min for GP2, about 16 min for GP3, and 17 to 18 min for GP1. The diphenyl column employed in this study separates and elutes tryptic glycopeptide according to the characteristics of the peptide, like the C18 reverse phase column commonly used in proteomics. Thus, even if the glycans were different, glycopeptides with the same peptide sequence were co-eluted at adjacent retention times. In addition, glycopeptides were somewhat separated according to the properties of the glycans even within the same peptide group under the diphenyl column [56]. On average, 16 and 23 glycopeptides were detected in the GP1 and GP3 groups, respectively. For GP2, since it contains two glycosylation sites, an average of 52 glycopeptides were identified. In all GP groups, most glycopeptides had sialylated N-glycans, of which the bi-antennary di-sialylated glycan was the most abundant (Figure 2(A-1,B-1,C-1)). Other N-glycopeptides containing undecorated or fucosylated/sialylated complex type glycans are not abundant, but are sufficiently identifiable in the magnified spectra (Figure 2(A-2,B-2,C-2)). In the deconvoluted spectrum, each peak represents a glycopeptide, and the spacing between adjacent peaks corresponds to the glycan residue difference. This clearly indicates that glycosylation within the same group is interrelated in the process of biosynthesis (Figure 2(A-3,B-3,C-3)).

Figure 3 shows a schematic diagram of site-specific glycosylation mapping of the haptoglobin purified from commercial sera. From the viewpoint of macroheterogeneity, glycosylation was the most abundant in GP3 and least detected in GP2. Bi-/tri-antennary sialylated complex types N-glycans were abundantly present in all glycosylation sites and mono-fucosylated glycans were also significantly observed, albeit in small amounts. Interestingly, the glycans of the glycopeptide are consistent with those obtained from the glycan profiling of the haptoglobin. Site-specific glycan mapping of the haptoglobin was implemented through the middle-up-down glycoproteome platform that provides information on glycan microheterogeneity and macroheterogeneity.

### 3.3. Gastric Cancer Biomarker Discovery via Middle-Up-Down Glycoproteome Platform

**Figure 4:**
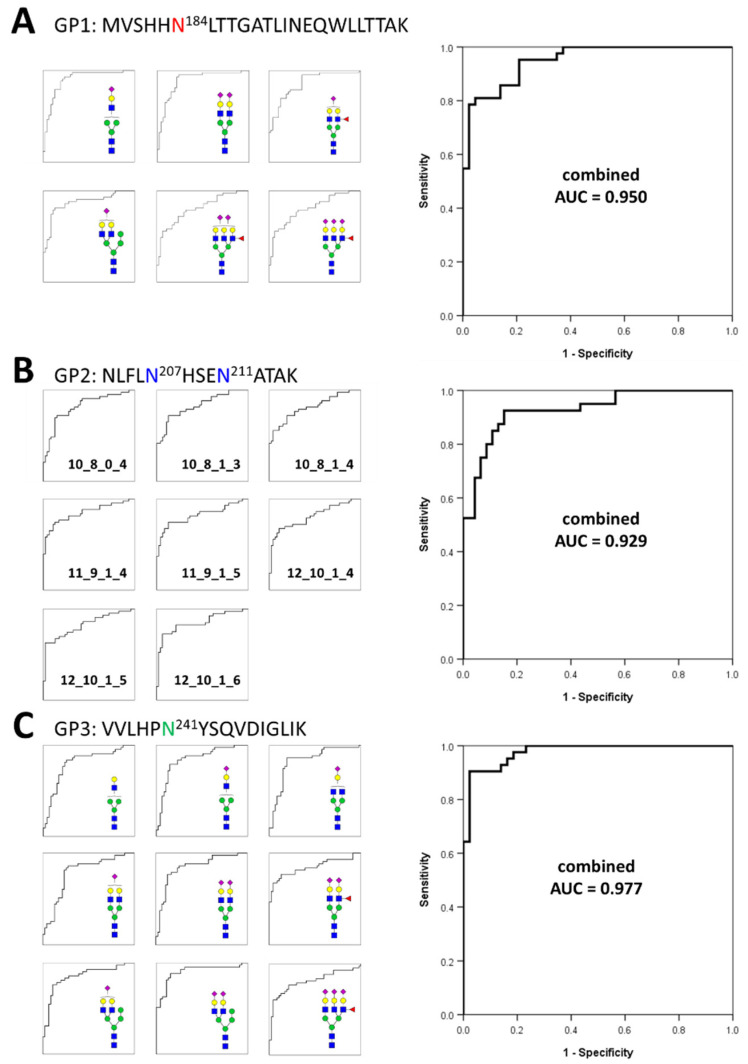
Individual and combined ROC curves using statistically significant glycopeptides. The small ROC curves on the left side are statistically significant individual glycopeptides in each glycopeptide group. On the right is the combined ROC curves derived using statistically significant individual glycopeptides for each glycopeptide group. The ROC curves of individual glycopeptides were calculated using their absolute abundances and combined ROC curves were derived using the logistic regression of glycopeptides. (**A**)—6 individual glycopeptides and their combined biomarker from GP1; (**B**)—8 individual glycopeptides and their combined biomarker from GP2; (**C**)—9 individual glycopeptides and their combined biomarker from GP3.

Based on the frequency and abundance of individual glycopeptides, we discovered potential biomarkers that could differentiate gastric cancer patients from healthy controls. Student’s *t*-test was performed using 71 glycopeptides detected with a frequency of 70% or higher in all the samples tracked in the glycan heterogeneity monitoring. Subsequently, significant glycopeptides were selected according to two criteria, the *p*-value (*p* < 0.00001) and frequency (more than 90% in all samples). A total of 23 potential biomarkers were determined from three different glycopeptide groups (Table 1). Each glycopeptide biomarker presented an area under the curve (AUC) of 0.783 to 0.901 in the receiver operation characteristic (ROC) analysis. As a result of classifying according to the glycopeptide group, 6 markers were found in GP1, 8 markers in GP2, and 9 markers in GP3. Interestingly, the markers with the highest and lowest AUC values all belonged to the GP3 group. From the number of glycopeptide markers found and the AUC values, it can be expected that the glycosite Asn241 present in GP3 is most associated with gastric cancer. To complement and improve the sensitivity and specificity of the markers, we applied a logistic regression model to potential biomarkers and calculated a combined ROC curve [57,58,59]. The AUC improved significantly to 0.950 (GP1), 0.929 (GP2), and 0.977 (GP3), respectively (Figure 4). In particular, the abundance of all potential glycopeptide biomarkers was increased in gastric cancer patients, and most of the markers had differences in monosaccharide residues, clearly indicating that they correlated with each other. Changes in the glycan compositions of the haptoglobin glycopeptide suggested that in gastric cancer, the composition of one specific glycan not only changes independently but also affects the glycan synthesis itself. Compared with previous haptoglobin studies for gastric cancer biomarkers [33], the middle-up-down approach simultaneously provides the middle-up-down approach simultaneously provides information on the microheterogeneity and macroheterogeneity of glycosylation, enabling the discovery of potential biomarkers with high sensitivity and high specificity.

**Figure 5:**
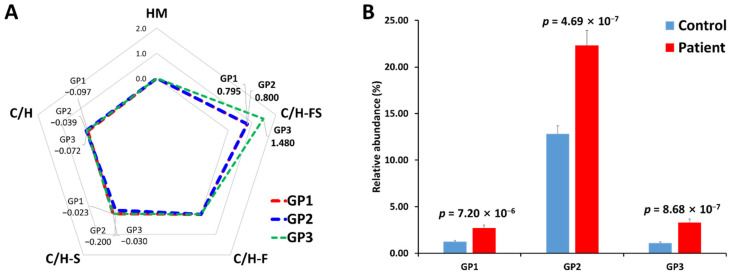
Comparison of N-glycosylation on haptoglobin between the healthy control and gastric cancer patient samples. (**A**) The radar chart of log_2_ fold changes (the ratio of cancer to control) for glycan classes of each glycopeptide group. (**B**) The relative abundance and *p*-value of glycoforms with both fucosylation and sialylation. HM—high mannose type; C/H—complex/hybrid type without fucose or sialic acid; C/H-S—complex/hybrid type with only sialic acid; C/H-F—complex/hybrid type with only fucose; C/H-FS—complex/hybrid type with fucose and sialic acid.

In addition, in order to verify the association between the glycan class of serum haptoglobin glycopeptides and gastric cancer, their expression levels were compared through a log_2_ fold change (Figure 5A). N-glycan can be classified into five biosynthetic groups: high mannose (HM) glycans; undecorated complex/hybrid (C/H) glycans; fucosylated complex/hybrid (C/H-F) glycans; sialylated complex/hybrid (C/H-S) glycans; and fucosylated-sialylated complex/hybrid (C/H-FS) glycans. The changes in the glycopeptides decorated with C/H-FS were particularly noticeable in all glycopeptide groups. The fucosylated/sialylated glycoforms increased regardless of the glycopeptide group, and the *t*-test also showed statistically significant values (Figure 5B).

Haptoglobin primarily binds to hemoglobin, but this binding is not related to glycans because the peptide sequence of hemoglobin subunits does not contain a glycosylation site. On the other hand, haptoglobin binds to at least four receptors on leukocytes, including CD163, CD22, CCR2, and CD11b/CD18. These ligand–receptor interactions are one of the major roles of glycosylation and are reported to be affected by fucosylation and sialylation [47,48]. The fucosylated and sialylated glycans of haptoglobin were identified in a previous study as sLe^x^ (sialyl-Lewis x) or sLe^a^ (sialyl-Lewis a) epitope [30], which is the terminal structure of fucosylation and sialylation. These sLe epitopes have been reported to be associated with cancer progression [48,60]. The high expression of fucosylation/sialylation in the haptoglobin of gastric cancer patients found in our study can also be expected to have a strong correlation with cancer progression. In addition, our findings show significant consistency with previous studies of glycosylation changes in haptoglobin, suggesting that the middle-up-down glycoproteome approach is sufficient to monitor haptoglobin glycosylation.

## 4. Conclusions

Glycosylation is of particular interest in the field of diagnostic biomarker research because it is highly sensitive to various diseases, especially cancer. In particular, research and applications related to glycosylation are accelerating with the advancement of mass spectrometry, a high-sensitivity and high-resolution instrument. Of the various glycoform characterization methods, in-depth information of glycosylation can be obtained from site-specific glycopeptide profiling using tandem MS, but multi-step sample preparation and complex data interpretation make it difficult to expand into clinical use. We developed a middle-up-down glycoproteome platform that can rapidly and accurately obtain the microheterogeneity and macroheterogeneity of a targeted glycoprotein without tandem MS analysis. Based on this platform, we were able to discover potential glycopeptide biomarkers with high sensitivity and high specificity that exhibit high AUC (0.783 to 0.901) at the molecular level required for actual clinical diagnosis. In addition, the biological association between specific glycosylation sites of haptoglobin and gastric cancer could be identified through glycopeptide grouping. This glycoproteome assay is a highly effective non-invasive platform that can be applied to the analysis of haptoglobin, as well as other glycoproteins with multiple glycosylation sites. Although marker validation using large-scale samples is required, this new analysis platform is expected to be widely used for biomarker discovery and clinical diagnosis based on a variety of targeted glycoprotein associated with diseases. In addition, the understanding of disease through biological association with changes in the glycosite of protein could be utilized for glycosylation-based drug development and personalized medicine.

## Figures and Tables

**Table 1:** The list of glycopeptides found in serum haptoglobin representing a significant difference between healthy controls and gastric cancer patients. Glycopeptides were selected based on the Student’s *t*-test (*p* < 0.00001). A complete list of glycopeptides is included in Table S4.

GP Group ^a^	Mass	N-Glycan Composition ^b^				*p*-Value	AUC
		Hex	HexNAc	Fuc	NeuAc		
GP1	4226.930	4	3	0	1	3.11 × 10^−11^	0.895
	4738.120	5	4	1	1	1.17 × 10^−8^	0.873
	4754.115	6	4	0	1	8.63 × 10^−11^	0.864
	4883.157	5	4	0	2	3.13 × 10^−11^	0.887
	5394.348	6	5	1	2	1.19 × 10^−7^	0.831
	5685.443	6	5	1	3	1.12 × 10^−6^	0.803
GP2	5722.234	10	8	1	3	3.28 × 10^−9^	0.828
	5867.271	10	8	0	4	8.18 × 10^−8^	0.818
	6013.329	10	8	1	4	2.32 × 10^−8^	0.814
	6378.461	11	9	1	4	9.32 × 10^−11^	0.848
	6669.557	11	9	1	5	6.78 × 10^−9^	0.820
	6743.593	12	10	1	4	1.74 × 10^−7^	0.805
	7034.689	12	10	1	5	1.86 × 10^−9^	0.823
	7325.784	12	10	1	6	7.50 × 10^−10^	0.846
GP3	3051.453	4	3	0	0	3.23 × 10^−7^	0.803
	3342.549	4	3	0	1	1.19 × 10^−8^	0.840
	3545.628	4	4	0	1	1.33 × 10^−7^	0.827
	3707.681	5	4	0	1	3.25 × 10^−6^	0.783
	3869.734	6	4	0	1	9.63 × 10^−9^	0.849
	3998.776	5	4	0	2	5.54 × 10^−9^	0.844
	4144.834	5	4	1	2	3.51 × 10^−9^	0.844
	4160.829	6	4	0	2	7.82 × 10^−12^	0.901
	4801.062	6	5	1	3	1.75 × 10^−6^	0.805

^a^ GP group refers to a set of glycopeptides having the same tryptic peptide sequence with different glycan moieties. The peptide sequence of GP1 is MVSHHN^184^LTTGATLINEQWLLTTAK, of GP2 is NLFLN^207^HSEN^211^ATAK, and of GP3 is VVLHPN^241^YSQVDIGLIK. ^b^ The monosaccharides of N-glycan composition represent Hex = Hexose; HexNAc = N-acetylhexosamine; Fuc = Fucose; NeuAc = N-acetylneuraminic acid.

## Supplementary Materials

The following are available online at https://www.mdpi.com/article/10.3390/jpm11060575/s1, Figure S1: Pearson correlation coefficient (*R*) of three glycopeptides of haptoglobin composition derived from 10 commercial serum samples. (a) GP1, (b) GP2, and (c) GP3, Table S1: Clinical information of samples involved in this study, Table S2: The list of 41 N-glycans of haptoglobin used for construction of in-silico glycopeptide library, Table S3: The list of in silico haptoglobin glycopeptide library, Table S4: A complete list of glycopeptides found in serum haptoglobin.
