# Supplementary material for: Detection of Aberrant Glycosylation of Serum Haptoglobin for Gastric Cancer Diagnosis Using a Middle-Up-Down Glycoproteome Platform
Source: J Pers Med. 2021 Jun 18;11(6):575. doi: 10.3390/jpm11060575 (PMC8235735; doi:10.3390/jpm11060575)
Supplement: Supplementary file 1 [file jpm-11-00575-s001.zip › Suppl_info.pdf]

Supplementary information

Detecting aberrant glycosylation of serum haptoglobin for gastric cancer diagnosis using middle-up-down glycoproteome platform

Seunghyup Jeong[1,2], Unyong Kim[3], Myung Jin Oh[1,2], Jihyeon Nam[1,2], Se Hoon Park[4], Yoon Jin Choi[5], Dong Ho Lee[5], Jae-Han Kim[6], and Hyun Joo An[1,2*]

1. Asia-pacific Glycomics Reference Site, Chungnam National University, Daejeon, Korea

2. Graduate School of Analytical Science and Technology, Chungnam National University, Daejeon, Korea

3. Biocomplete Inc., Seoul, Korea

4. Division of Hematology-Oncology, Department of Medicine, Sungkyunkwan University Samsung Medical Center, Seoul, Korea

5. Department of Internal Medicine for Gastroenterology, Seoul National University Bundang Hospital, Seongnam, Korea

6. Department of Food and Nutrition, Chungnam National University, Daejeon, Korea

Correspondence:

Hyun Joo An, Graduate School of Analytical Science and Technology, College of Engineering II, Chungnam National University, 99 Daehak-ro, Yuseong-gu, Daejeon 34134, Republic of Korea. E-mail: hjan@cnu.ac.kr

Supplementary information

Table of Contents

Figure S1. Pearson correlation coefficient (*R*) of three glycopeptides of haptoglobin composition derived from 10 commercial serum samples. (a) GP1, (b) GP2, and (c) GP3.

Table S1. Clinical information of samples involved in this study.

Table S2. The list of 41 *N*-glycans of haptoglobin used for construction of *in-silico* glycopeptide library.

Table S3. The list of *in-silico* haptoglobin glycopeptide library.

Table S4. A complete list of glycopeptides found in serum haptoglobin.

Figure S1. Pearson correlation coefficient (*R*) of three glycopeptides of haptoglobin composition derived from 10 commercial sera samples. (A) GP1, (B) GP2, and (C) GP3.

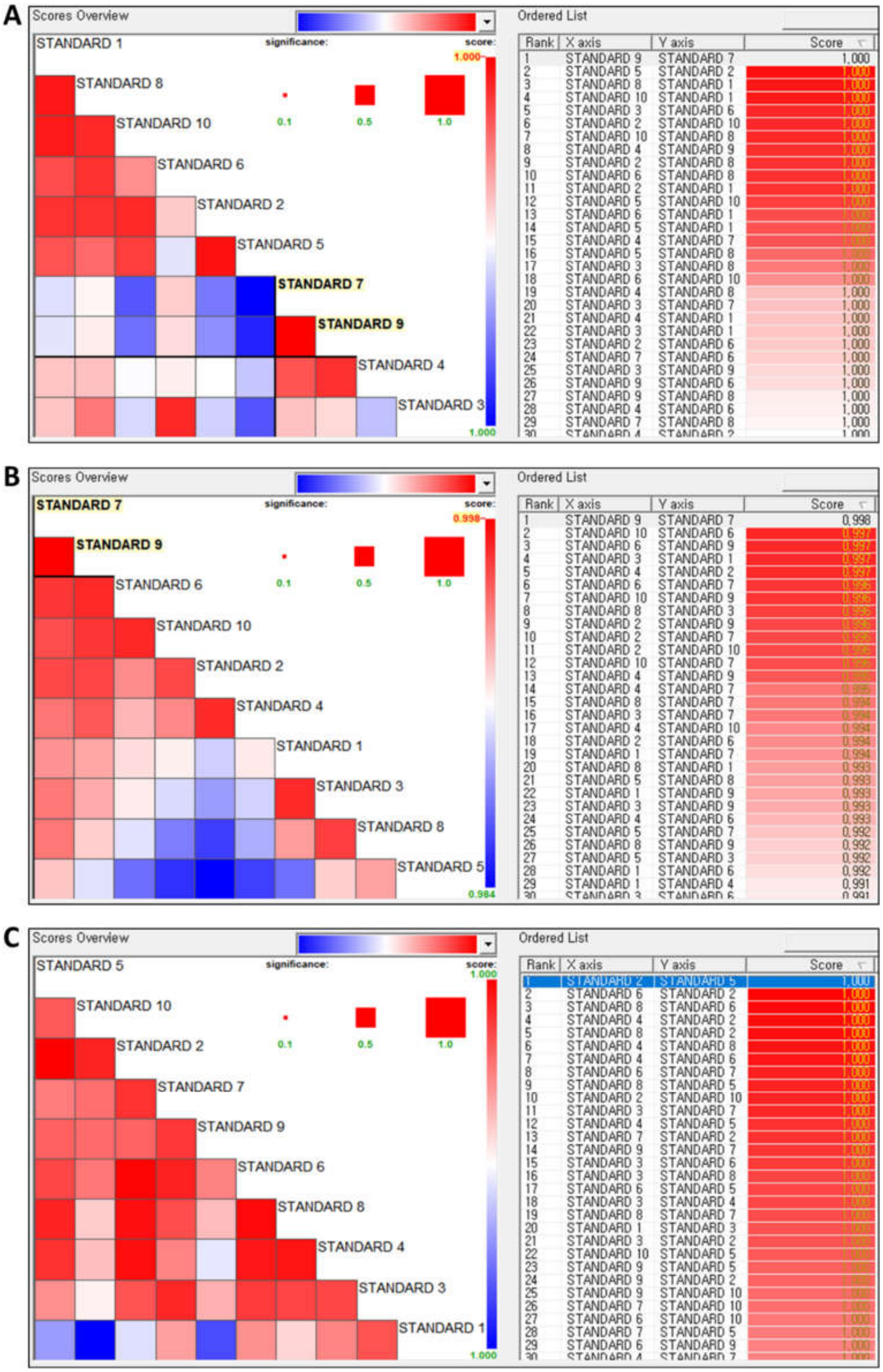

Table S1. Clinical information of samples involved in this study.

**Information**		**Healthy Control (*n* = 47)**	**Gastric Cancer (stage IV, *n* = 43)**
**Age (years)**	Median (range)	55 (31 - 76)	44 (33 - 77)
**Gender**	Male	9	25
	Female	38	15
